# A novel strategy for isolation of mice bone marrow endothelial cells (BMECs)

**DOI:** 10.1186/s13287-021-02352-3

**Published:** 2021-05-03

**Authors:** Alhaji Osman Smith, Seyram Yao Adzraku, Wen Ju, Jianlin Qiao, Kailin Xu, Lingyu Zeng

**Affiliations:** 1 Blood Diseases Institute, Xuzhou Medical University, Xuzhou, 221002 China; 2 Key Laboratory of Bone Marrow Stem Cell, Xuzhou, 221002 Jiangsu Province China; 3 Department of Hematology, The Affiliated Hospital of Xuzhou Medical University, Xuzhou, 221002 China

**Keywords:** Isolation, Primary bone marrow endothelial cells (BMECs), Tumor necrosis factor-alpha (TNF-α)

## Abstract

**Background:**

In the bone marrow microenvironment (BM), endothelial cells are individual cells that form part of the sinusoidal blood vessels called the “bone marrow endothelial-vascular niche.” They account for less than 2% of the bone marrow cells. They play essential functions by generating growth and inhibitory factors that promote the hematopoietic stem cells (HSCs) regulation. In response to inflammatory stimuli, the BMECs increase in proliferation to maintain the blood vessels’ integrity within the BM. The inflammatory response releases cytokines such as tumor necrosis factor-alpha (TNF-α) that promote vascular endothelial cells’ expansion and upregulation of adhesion molecules (ICAM-1 and VCAM-1, respectively) in the BM. However, the evaluation of mouse BMECs in the bone marrow microenvironment is scared by a lack of mouse bone marrow endothelial cell primary culture

**Methods:**

Two steps approach for isolation of bone marrow endothelial cells (BMECs) from mice. In brief, the bone marrow cells extracted from the mice long bones were cultured overnight with Dulbecco’s modified Eagle’s medium (DMEM) supplemented with 20% fetal bovine serum (FBS) and antibiotics to separate between marrow-derived adherent and non-adherent cells. The floating cells were discarded, and the adhered section detached with accutase and BMECs selected using CD31 microbeads. The isolated BMECs were cultured in a dish pre-coated with rat-tail collagen type 1 with endothelial cells medium supplement with growth factors. The cells were verified by confocal microscopy for morphology and tube formation by matrigel assay. We validate the cells’ purity by flow cytometry, RT-qPCR, immunofluorescence staining, and immunoblotting by established BMEC markers, PECAM-1, VE-cadherin, vascular endothelial cell growth factor receptor-2 (VEGFR2), CD45, E-selectin, and endothelial selectin adhesion molecule (ESAM). Lastly, we characterize BMEC activation with recombinant TNF-α.

**Results:**

Our method clearly defined the cells isolated have the characteristics of BMECs with the expression of CD31, VE-cadherin, E-selectin, VEGFR-2, and ESAM. The cells’ response to TNF-α indicates its inflammatory function by increasing proliferation and upregulation of adhesion molecules.

**Conclusions:**

This study outline a simple new technique of isolating mouse BMEC primary culture and a suitable method to evaluate the function and dysregulation of BMEC in in vitro studies using mouse models.

**Supplementary Information:**

The online version contains supplementary material available at 10.1186/s13287-021-02352-3.

## Background

The endothelial cells are part of the sinusoid-vascular niche in the bone marrow microenvironment. They play a significant role in producing growth and inhibitory cytokine that regulates the function of the hematopoietic stem cells. Also, they display adhesion molecules that interconnecting the hematopoietic progenitor cells. In the bone marrow microenvironment, they line the lumen of the sinusoidal-vascular niche. In response to inflammatory stimuli, they increase in proliferation to maintain the integrity of the blood vessels. The inflammatory response releases cytokines that promote their activation, such as tumor necrosis factor, interleukin 6. These cytokines, in turn, increase the expression of the endothelial adhesion molecule, such as vascular adhesion molecules (VCAM-1), E-selectin, and intracellular adhesion molecules (ICAM-1), respectively [1–3]. Recent studies have documented that the endothelial niche is split into the sinusoidal place and vascular niche. They are both acknowledged by the positive marker for CD31 and vascular endothelial adhesion molecule (VE-cadherin), but the sinusoidal slot expresses positive attributes for vascular endothelial growth receptor factor 2 (VEGFR-2) [4–7]. Besides, most studies have documented that endothelial cells express a positive marker such as von Willebrand factor (VWF), intracellular adhesion molecule 1 (ICAM-1 /CD106), vascular adhesion molecule 1 (VCAM-1/CD105), E-selectin, BMA120, and endothelial selective adhesion molecule (ESAM) [8–10]. Endothelial cell dysfunction within the bone marrow environment due to chemotherapeutic agents or radiotherapy may result in the deletion of VEGFR2 in adult mice. It may prevent the renewal of the sinusoidal endothelial and the recovery of hematopoietic stem cells [11]. There are different endothelial cells, such as human umbilical vein endothelial cells (HUVECs) and mouse brain-derived endothelial cells (MBDECs). Endothelial cells in various locations exhibit similar functions, such as recruiting progenitor cells in response to inflammatory stimuli and capillary-like lumen tube formation [12]. Several procedures have been illustrated in the literature (Table 1) [13–17] to isolate endothelial cells from the bone marrow. The one most commonly used is fluorescence-activated cell sorting (FACS) with antibodies against bone marrow endothelial cells such as CD31 (PECAM-1) and CD45. However, this method is costly and requires dedicated, well-trained personnel to ensure reliable cell count, longer processing time, and limited FACS antibody reagents. However, the studies of mouse bone marrow endothelial cells are limited by a lack of primary bone marrow endothelial cells. A suitable method to clearly define mouse endothelial cells’ function in vitro hindered the genetics, phenotypic, or proteomic studies using mouse models as described in the Yuxin Feng article [16]. This study developed a strategic approach for isolating mouse bone marrow endothelial cells by cultured, marrow-derived adherent cells, including vascular endothelial cells, fibroblast, and mononuclear phagocytes. The adherent marrow-derived cells were then suspended with a single magnetic MLECs CD31 microbead incubation and bounded cell isolation to separate the vascular endothelial cells from the rest of the adherent cells. We hypothesized that this method could evaluate the primary mouse bone marrow endothelial cells in vitro studies and better understand the genetics, phenotype, or proteomic studies using a mouse model. We will also highlight factors that need to take into consideration when using this method.

**Table 1:** Published methods for isolation of endothelial cells from the bone marrow

References	Type of species	Method of isolation	Purification method
Peter A G McCourt [13]	Mouse	Mechanically digestion of bone marrow cells	Magnetic beads labeling Advanced glycation end-products (AGE)/fluorescence-activated cell sorting (FACS)
S Irie [14].	Rat	Collagenase digestion of bone marrow cells	Discontinuous gradient centrifugation on Percoll (densities 1.04 and 1.06)
Nakatsuka, Ryusuke [15]	Murine	Enzymatic digestion of murine bone	Fluorescence-activated cell sorting (FACS)
Yuxin Feng [16]	Mouse	Not available	Fluorescence-activated cell sorting (FACS)
Van Beijnum, Judy R. Rousch, Mat [17]	Human/other species.	Mechanically minced and enzymatic digestion with collagenase and dispase	Single magnetic beads cell suspension/ high-speed cell sorting

## Materials and methods

### Materials

1. Dulbecco’s phosphate buffer solution (DPBS) potassium chloride (KCl) 0.2g/L, sodium chloride (NaCl) 8.0g/L, sodium phosphate dibasic (Na_2_HPO_4_) 1.15g/L potassium phosphate monobasic (KH_2_PO_4_) 0.2g/L in Milli-Q water [pH 7.2–7.6 adjusted with hydrochloride acid (HCl) sterilized and stored at 4°C.
2. 0.5-M ethylenediaminetetraacetic acid (EDTA) sterile by autoclave store at 4°C.
3. Dulbecco’s modified Eagle’s medium (DMEM) and fetal bovine serum (FBS) (Gibco, Thermo Fisher Scientific).
4. 0.5-ml Nest micro-centrifuge tube and 1.5-ml Eppendorf centrifuge tube, 18-G needle and syringe, 70-μm cell strainer (Biologix group limited), sterile 15-ml and 50-ml conical tube, and 70% ethanol
5. Accutase solution (Absin, Biochemical Company)/trypsin/ETDA (VICMED 0.25% 0.02%)
6. 10-mm cell culture dish, 12–48-well plates.
7. Endothelial cell medium (EBM-2), [Lonza, Clonetics®] supplement with growth factors: 25-ml fetal bovine serum (FBS), 0.2-ml hydrocortisone 2ml, and 0.5ml of human fibroblast growth factor (hFGF), vascular endothelial growth factor (VEGF), human epidermal growth factor (hEGF) ascorbic acid heparin, gentamicin/amphotericin.
8. 10,000uints/ml)/streptomycin 10,000ug/ml (Gibco, Thermo Fisher Scientific).
9. Micro-dissecting board, surgical scissors, sterile gloves, and gauze.
10. Rat-tail collagen type 1 3mg/ml (Catalog #A10483-01, Thermo Fisher Scientific) plus 0.2M sterile HCL
11. CD 31 microbeads, MS column (MACS, Miltenyi Biotec).

### Equipment

1. Laminar flow hook (Thermo Fisher Scientific)
2. High- and low-speed centrifuge (BECKMAN COULTER, Microfuge®20R, and serial no. MRZ14H028, made in Germany, and AIRTECH-KDC-40 Anhui, serial number 02241700049)
3. Nikon Eclipse microscopy (Nikon Eclipse Ti microscopy, Tokyo, Japan)
4. Confocal microscopy (Airyscan, Germany, serial number 182412128)
5. Corning® Matrigel® Basement membrane matrix, Phenol Red-free. *LDEV-free
6. Flow cytometry (BD LSRFortessa™)
7. 5% CO_2_ humidified incubator (HERA CELL 150i, CO_2_ incubator, Thermo Fisher Scientific, SERIAL NO. 41629032, Made in Germany)
8. Autoclave machine (HIRAYAMA, HICLAVE™ HVE-50, serial number 30613065826, Made in Japan)
9. Roche real-time quantitative machine (Photonic A-1160, CH-6343)and LightCylcer® 480 Software release 1.5.1.62 SP2)
10. Bio-Rad agarose electrophoresis machine(PowerPac™ Basic serial number 041BR142780, made in Singapore)
11. Magnetic separation column ((MACS, Miltenyi Biotec)
12. NANODROP 2000c Spectrophotometer (Thermo Fisher Scientific)
13. Polymerase chain reaction machine (Mastercycler Nexus gradient, Eppendorf serial number 6331ER910944, Made in Germany)

### Pre-requisite solution

1. 1-mm ethylenediamine tetraacetic acid (EDTA)/DBPS (100ul of 0.5M EDTA in 50ml DPBS)
2. Dulbecco’s modified Eagle’s medium (DMEM) plus 20% fetal bovine serum (FBS), and 500ul of penicillin (10,000uints/ml)/streptomycin 10,000ug/ml
3. 50μg/cm^2^ rat tail collagen type 1 in 0.02M HCl, pre-coat the cell culture dish (12–48 well plates) overnight in the incubator at 37°C
4. Prepare an appropriate volume of the endothelial cells medium supplement with growth factors (minimum 50ml) store at 4°C for 1 month
5. Harvesting buffer solution (2% FBS/1mm EDTA/DBPS) store at 4°C for 1 month

### Animal model

The wild-type C57BL/6 mice (age 8–12 weeks) were obtained from the Jackson laboratory. The mice were housed per the guidelines of the Xuzhou Medical University, Jiangsu Province, China. The use committee approved the Xuzhou Medical University’s animal studies (Xuzhou, China) and the National Institute of Health guide for the care and use of laboratory animals, Jiangsu Province, China (Acceptance number: XZMC20130226).

#### Day 1

##### The step-by-step process of bone marrow extraction

1. Mice were aged 8–12 weeks (number of mice per experiment (*N*) = 6) terminated by cervical dislocation. The whole mice were soaked in 70% ethanol for 2–5 min, then placed on the sterile dissecting board on the laminar flow hook. The long bones of the 12 femora and 12 tibias were pulled off with micro-dissecting scissors placed in sterile Dulbecco’s phosphate buffer solution (DPBS)
2. The muscles were detached from the bones by forceps, and the bones scrubbed to remove any residual soft tissues. The bones were washed twice with DPBS solution containing 1mmEDTA. One edge of the long bones cut and placed in a new 10-mm dish containing the DPBS/EDTA solution.
3. An 18-G needle was pushed to the bottom of the 0.5-ml Nest microcentrifuge tube. The long bones’ edge was cut and inverted downwards in the 0.5-ml Nest micro-centrifuge (maximum of 2 tibias and 2 femora) and the lid closed.
4. The 0.5-ml Nest micro-centrifuge tube was transferred into 1.5-ml Eppendorf tubes sealed with Parafilm and centrifuge at 15,000*g* for 30 s. The 0.5-ml Nest microcentrifuge was discarded and the visible pellet at the bottom of the 1.5 ml Eppendorf tubes suspended with sterile DPBS/EDTA solution.
5. The suspended bone marrow cells with the PBS/EDTA solution, filtered with a 70-μm cell strainer (Biologix group limited) and centrifuge at 300*g* for 5 min at room temperature. The pellet resuspended in DMEM supplemented with 20% fetal bovine serum (FBS), and 500μl of penicillin (10,000 units/ml)/streptomycin 10,000ug/ml.
6. The cells were plated in a sterile 10-mm dish with a minimum of 10^9^ cells/plate and incubated in a 5% CO_2_ humidified incubator overnight.

#### Day 2

##### The step-by-step process of isolating bone marrow endothelial cells

1. After overnight incubation, the floating cells poured off. The cultured marrow-adherent cells were washed with sterile DPBS twice and detached with appropriate accutase solution for 15 min at room temperature.
2. The detached cells were suspended with the harvesting buffer [PBS, 2% FBS, 1-mm ethylenediaminetetraacetic acid (EDTA), and penicillin/streptomycin)] with repeated pipetting to detached the remaining cells. The cells were transferred into 15-ml tubes, centrifuge at 300*g* for 10 min at room temperature—the total cell number determined by a hemocytometer or automated cell counting machine.
3. The pellet cells were resuspended with the harvesting buffer solution with CD31 microbeads by manufacturer’s protocols and kept at 4°C for 15 min.
4. The cells were resuspended with 10 ml of harvesting buffer solution, then centrifuge at 300*g* for 10 min at room temperature to wash off the cells’ excess beads after 15 min incubation.
5. The MS column attached to the magnetic washed once with the harvesting buffer, bone marrow cells pass through the column. The magnetic cells were washed three times with the harvesting buffer, followed by a single wash with endothelial cell medium supplement with growth factors.
6. The magnetic cells are pushed into new sterile tubes with the endothelial cell medium supplement with growth factors; the total number of cells count is determined by a hemocytometer or automated cell-counting machine.
7. The plate pre-coated with rat-tail collagen type 1, washed with sterile DPBS solution twice, and followed by one wash with the endothelial cell medium supplement with growth factors. Appropriate cells number (200–300 cells/ml) seeded into the coated dishes (12–48-well plates) and incubated into the incubator.

#### Characterization of primary bone marrow endothelial cells (BMECS)

##### Bone marrow endothelial cell structure visualization

The cultured cells sequentially observed every day for the capillary-like structure appearance of endothelial cells with confocal microscopy and photographed (Nikon Eclipse Ti microscopy, Tokyo, Japan).

##### Matrigel capillary tube formation assay of BMECs

Matrigel was allowed to thaw on ice overnight according to the manufacture’s protocols. Prechilled 24-well plates were coated on ice with 200-μl Matrigel per well. Gels were incubated for 30 min for solidification at 37°C. BMECs 10^5 cells, resuspended in 500μl of the pre-cold endothelial cell medium supplemented with growth factors, and cells plated on the gels. The plated cells were incubated in the humidifier at 37°C for 4–7 days. The morphological changes are periodically monitored and photographed.

##### Flow cytometry analysis of bone marrow endothelial cells

The selected CD31 microbead-positive cells analyzed by flow cytometry with a panel of antibodies: [CD31 (PE. anti-mouse, eBioscience™), CD45 (FITC anti-mouse, eBioscience™) CD106 (PE, Rat-anti-mouse, BD-Pharmingen™), CD144 (APC, anti-mouse, eBioscience™), and endothelial selective adhesion molecule (ESAM) (APC, anti-mouse, Biolegend®) antibodies]. 500,000 cells/ml were collected per tube, washed with PBS, centrifuged at 400*g* for 5 min at room temperature (×2), incubated with the recommended dilution of antibodies, stored at 4°C for 1 h, and analyzed by flow cytometry (BD LSRFortessa™) within 24 h. The lower threshold uses to exclude debris and the live cells with gating (20,000 cells) according to forward scatter (FSC) × side scatter (SSC), followed by sections containing the antibodies. The data retrieve from the flow cytometry software and analyze by FlowJo software version 7.6.2.

##### Characterization of primary bone marrow endothelial cells by real-time quantitative PCR (RT-qPCR)

To verify the molecular expression of the bone marrow endothelial cells, total RNA extracted from the cells after 7 days of incubation, and the CD31 microbead-negative cells immediately after isolation using Trizol reagents (TIANGEN Cat#dp424). The cDNA is synthesized using 5X. All in one RT Master Mix (Cat.No.G492) and kept at −20°C until ready for use. Primer sequences and probes are shown in (Table 2). For RT-qPCR, the synthesized cDNA samples 10 ng were amplified with the SYBR green master mix in a final volume of 20 ul, as described in our previously published article [18]. The mean threshold values are used to evaluate the molecular gene expression with normalization with mouse beta-actin and GAPDH.

**Table 2:** Quantitative PCR primers sequences

Target genes	→Forward 5’ 3.’	→Reverse 3 ’ 5.’
**BETA ACTIN**	**ATGTGGATCAGCAAGCAGGA**	**AAGGGTGTAAAACGCAGCTCA**
**ESAM**	**CCTGGTCAGTAGCCTGGTTC**	**CCTGGTCAGTAGCCTGGTTC**
**VE-CADHERIN**	**TTTGCCCAGCCCTACGAACC**	**ACCGCCGTCATTGTCTGCCT**
**ICAM-1**	**GGCATTGTTCTCTAATGTCTCCG**	**GGCATTGTTCTCTAATGTCTCCG**
**VCAM-1**	**GGCATTGTTCTCTAATGTCTCCG**	**CTCCTTCACACACATAGACTCC**
**GAPDH**	**CATGGCCTTCCGTGTTCCTA**	**GCGGCACGTCAGATCCA**

##### The validation of bone marrow endothelial cells by immunoblotting analysis

Four hundred thousand cells (400,000 cells) were placed in a plate then incubate for 7 days. A herpes-chap lysis buffer containing the protease inhibitors pour into the dish, set for 10 min, and adherent cells scraped off with a cell scraper. The lysed cells were centrifuged at 20,000*g* for 30 min at 4°C. While for CD31 microbead-negative selected cells, the protein was isolated immediately after isolation and kept at −20°C until ready for use. The cell supernatants (20 μg of proteins) run on SDS-PAGE 8–12% gel (BIO-RAD; Hercules, CA). The proteins were transferred to the P 0.45 PVDF blotting membrane (Amersham™Hybond™ Germany) by the wet transfer method. Primary and secondary antibodies are shown in (Table 3) with a dilution of 1:1000 for primary antibodies and 1:5000 for secondary antibodies in 5%BSA/TBST solution.

**Table 3:** Western blot and immunofluorescence staining antibodies

Antigen	Ab species	Company	Cat. number
RABBIT Ig-HRP	**Goat**	**Absin**	**AS001**
MOUSE Ig-HRP	**Goat**		
GAPDH	**Rabbit-polyclonal**	**Service bio**	**GB1100**
E-SELECTIN	**Mouse monoclonal**	**Santa Cruza Biotechnology**	**G2718**
VE-CADHERIN (CD144)	**Rabbit-polyclonal**	**Affinity Bioscience**	**AF6265**
PECAM-1 (CD31)	**Rabbit-polyclonal**	**Affinity Bioscience**	**AF6191**
ICAM-1 (CD106)	**Rabbit-polyclonal**	**Affinity Bioscience**	**AF6088**
VCAM-1 (CD105)	**Rabbit-polyclonal**	**Affinity Bioscience**	**DF6082**
**VEGFR2 (FlK-1)**	**Rabbit-polyclonal**	**Bioworld Technology, Inc.**	**Q1169**
**Alexa Fluro®594** **Alexa Fliro®488**	**Goat Anti-rabbit (IgG** **Goat Anti-rabbit (IgG)**	**Abcam (Cambridge, MA)**	**Ab15008** **Ab15007**

##### The characterization of bone marrow endothelial cells by immunofluorescence staining

To certify the bone marrow endothelial cells for the expression of CD31 (PECAM-1), VE-cadherin (CD144) and ICAM-1 with passage zero were determined by direct immunofluorescence staining. The CD31 microbead-positive cells (50 000 cells) were plated into pre-coated 48-well with rat tail collagen type 1 for 5–7 days, as described above. After 60–70% of the confluence, the medium removed, cells fixed with 4% paraformaldehyde (PF) for 20 min at RT, followed by two wash 3 min apart with PBS. The cells were permeabilized with 100% cold methanol at room temperature for 20 min, rinsed with PBS three times, and blocked with 1% BSA/PBS for 1 h at room temperature. Cells incubated with the recommended dilution of primary antibodies overnight at 4°C. The cells were cleaned twice with PBS and counterstained with Hoechst for 5 min at 37°C. The cells were washed with PBS and imaging acquired using inverted Nikon microscopy (Nikon Eclipse Ti microscopy, Tokyo, Japan). Primary and secondary antibodies are shown in (Table 3) with a dilution of 1:100 for primary antibodies and 1:500 for secondary antibodies in 1%BSA/PBS solution.

#### Induction of primary bone marrow endothelial cells by recombinant tumor necrosis factor-alpha (TNF-α)

##### Assessment of primary bone marrow endothelial cell proliferation

To examine the cell proliferation of primary bone marrow endothelial cells, 10^5^cells were seed in 48-well plates incubated for 7 days. After 7 days, the BMECs were stimulating with recombinant TNF-α (10 ng/ml) and the control with 1% FBS with DPBS for 48 h. The medium containing the recombinant cytokine and FBS/DPBS was replaced with a new endothelial cell medium supplement with growth factors and incubated for another 7 days. The cells were harvested by trypsin/ETDA (VICMED 0.25% 0.02%), centrifuged at 350*g* for 5 min, wash 2x with PBS, and fixed with 70% cold–ethanol kept at −20°C for 1 h. The fixed cells were centrifuged as above followed by washed with FACS buffer incubated with Ki-67 (FITC, anti-mouse, BioLegend®) and F4/80 (APC, anti-mouse as isotype control BioLegend®) for 30 min at room temperature. The data was acquired by flow cytometry. The cells were stained with trypan blue for the cell count, and live cells were counted by hemocytometer.

##### The initiation of bone marrow endothelial cells by recombinant tumor necrosis factor-alpha

The molecular expression of markers specific for bone marrow endothelial cells verified by real-time quantitative polymerase chain reaction (RT-qPCR) or immunoblotting and immunofluorescence staining. The cells were cultured for 7 days to form a confluence, then stimulated with TNF-α at (10 ng/ml) and vehicle control (2% fetal bovine serum in PBS) for 48 h. Cells were then harvested for RT-qPCR and western blotting analysis and stained for immunofluorescence. All samples are done in triplicate (except immunoblotting, two experimental repeats), and results are expressed in the mean with standard deviation.

### Statistical analysis

All data were statistically analyzed using Graph Prism 6 and paired two-tailed student’s test used for comparison mean ± standard deviation. *P* values ≤ 0.05 are considered statistically significant.

## Results

### Bone marrow-derived adherent cell dissociation for isolation of BMECs

We used two different enzymatic digestion [accutase & trypsin/EDTA (0.25% 0.02%) solution] to dissociate the cultured bone marrow-adherent cells, followed by a single magnetic CD31 microbead suspension. For each enzymatic dissociation, six [6] mice per group (12 femora & 12 tibias), accutase, appeared to be less toxic than trypsin/EDTA solution with more than 1.5 × 10^6^ cells yield compared to trypsin/EDTA, which is less than 1.0x10^6^ cells. This data indicates that accutase does not damage the vascular endothelial cells’ encoded protein [19–21]. However, trypsin/ETDA is more effective in dissociating the bone marrow endothelial cells after several cultured days, but data is not provided.

### Morphological observation and tube formation of bone marrow endothelial cells

**Fig. 1:**
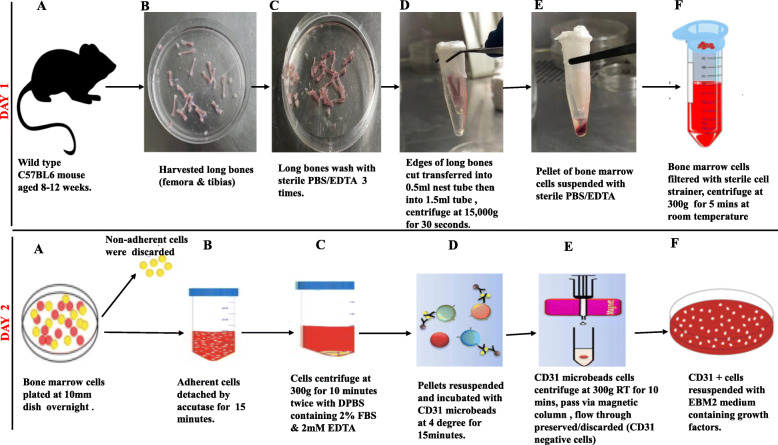
Step-by-step process of isolating bone marrow endothelial cells: Day 1: **a** the mice were killed by cervical dislocations soak in 70% ethanol for 2 min. **b** The long bone of femora and tibias harvested was placed in a sterile 10-mm dish after dissecting the muscle, tendon, and ligaments. **c** The long bones were washed with DPBS/EDTA solution three times. **d** One end of the long bone cut-off with dissecting forceps inserted into the 0.5-ml Nest microtubes was centrifuge at 15,000 for 30 s. **e** The pellet of the bone marrow after centrifugation (**f)** The bone marrow pellet was suspended with DPBS/EDTA solution, filtered with a cell strainer, and centrifuged at 300*g* for 10 min at room temperature. Day 2: **a** the bone marrow cells resuspended with DMEM supplemented with 20% FBS plus antibiotics and incubated in the incubator overnight. Non-adherent cells were discarded, and marrow-adherent cells were washed twice with DPBS/EDTA solution. **b** Accutase solution poured into the dish to detach the attached cells for 15 min at room temperature. **c** The cells were resuspended with DPBS/EDTA solution and centrifuged at 300*g* for 10 min. **d** The resuspended bone marrow cells were incubated at 4°C with the CD31 microbeads. **e** The beads pass via the column and wash three times. **f** The magnetic cells resuspended with endothelial cell medium supplement with growth factors

**Fig. 2:**
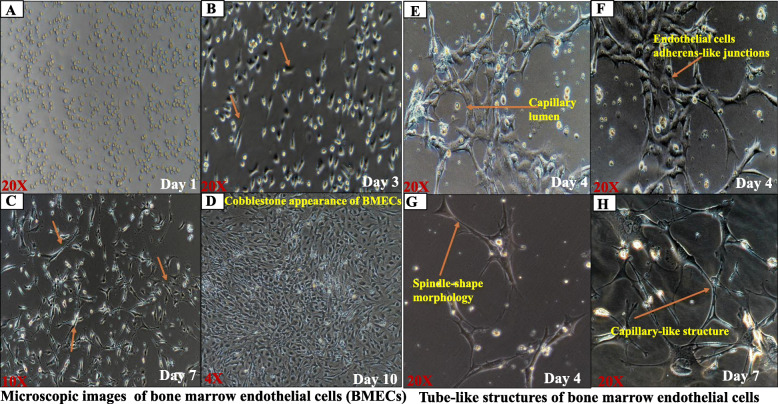
Morphology and tube formation of bone marrow endothelial cells. **a** Appearance of the cells after 24 h of isolation **(b/c** (20X). **b** Visualization of the ameboid-like structure of the bone marrow endothelial cells **(c)** appearance of the spindle-shaped morphology. **d**/4X Cobblestone appearance of the bone marrow endothelial cells after 100% confluence. **e**–**h**/20X Tube-like structure of bone marrow endothelial cells on Matrigel assay. **e** Capillary lumen appearance. **f** Endothelial cells like adherens junction with capillary lumen and tubing of the cells (**g**) spindle-like shape morphology and tubing of the cells. **h** Capillary-like structures of bone marrow endothelial cells on day 7. Three experiment repeats with 2 mice per group (4 femora and 4 tibias each)

In brief, after isolation of the bone marrow endothelial cells, as shown in Fig. 1. The 12-well plates pre-coated with 50μg/L with rat-tail collagen type 1 overnight in the humidifier incubate at 37°C. The pre-coated dishes were washed twice with sterile DPBS and rinse once with endothelial cell medium supplement with growth factors. The isolated cells culture in the 12-well plates is observed daily for the spindle shape’s appearance with the invert light microscopy. On the third day, the ameboid-like form is monitored, and after proliferation on day 7, the bone marrow endothelial cells assumed the spindle-shaped morphology (with 20x lens power) and cobblestone-like appearance after 100% confluence(with 4x lens power) shown in Fig. 2c, d. For bone marrow endothelial cell tube formation, the 24-well Prechilled on ice and pre-coated with matrigel solution. The pre-coated well was incubated at 37°C for 30 min. One hundred thousand cells are cultured into the matrigel basement membrane and are observed daily for the cells’ capillary-like tube formation. The bone marrow endothelial cells’ capillary-like lumen, endothelial cell adherens-like junction, and tube formation followed on a fourth day ( with 20x lens power), as shown in Fig. 2e–g. The bone marrow endothelial cells’ capillary-like structures were observed on the seventh day (with 20x lens power), as shown in Fig. 2h. These in vitro findings are bone marrow endothelial cells’ characteristics like other endothelial cells isolated from different tissues*.*

### Flow cytometry analysis of bone marrow endothelial cells

**Fig. 3:**
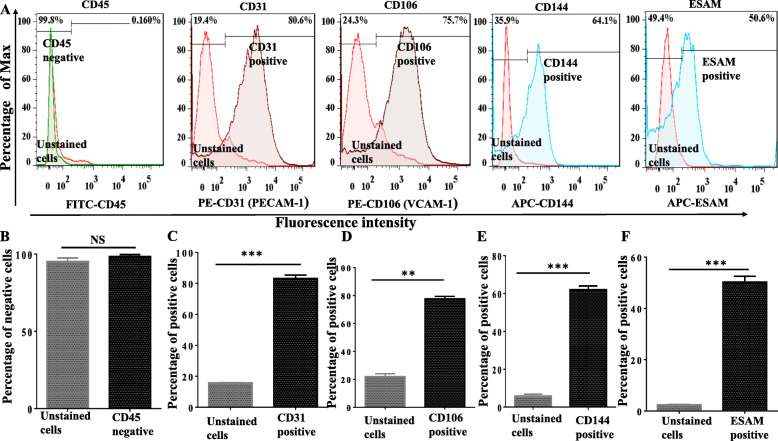
Characterization of primary bone marrow endothelial cells by flow cytometry: **a** the primary BMECs were negative for CD 45 (98.2%). **b** CD 31 (80.6%), **c** CD106 (7576%), **d** CD144 (64.4%), and **e** endothelial selective adhesion molecules (ESAM) (50.6%). The red tented histograms represent the unstained cells as the isotype control, and the colored histograms indicate the antigen staining of the endothelial cell’s surface markers. The bar chart indicates the mean ± SD using Student’s *t* test of two experimental repeats compared to the isotype control [*N* =2 with 6 mice/group (12 femora and 12 tibias)] and *p* value ≤ 0.05 and considered statistically significant. For CD 45, there is no statistical significance between the negative isotype control (unstained cells) and the stained cells, which verify the purity of the cells isolated are the characteristics of bone marrow endothelial cells, as endothelial cells express CD45-negative molecule. **p<0.01, ***p<0.001, and NS not significant

Briefly, the floating cells were discarded after overnight incubation, and the marrow-adherent cells were detached with accutase solution. The resuspended cells were centrifuged, and the bone marrow endothelial cells were selected with CD31 microbeads. The CD31-positive selected cells were stained for surface markers specific for endothelial cells (CD31, CD106, CD144, ESAM, and CD45^-^, respectively). The unstained cells were used as the negative isotype control, and the percentage of positive staining cells was presented in the histogram, as shown in Fig. 3a. The bone marrow endothelial cells express >98% of CD45-negative and CD45-positive expression for CD31 (83.13±2.196), CD106 (77.60±1.755), CD144 (62.0.±02), and ESAM (50.30±2.265) respectively compared to the negative isotype control (unstained cells) as shown in Fig. 3b–f. The characteristics expression of these surface markers by flow cytometry are highly specifics for endothelial cell detection. This data indicates that the cells isolated are precisely bone marrow endothelial cells.

### Real-time quantitative PCR and immunoblotting

**Fig. 4:**
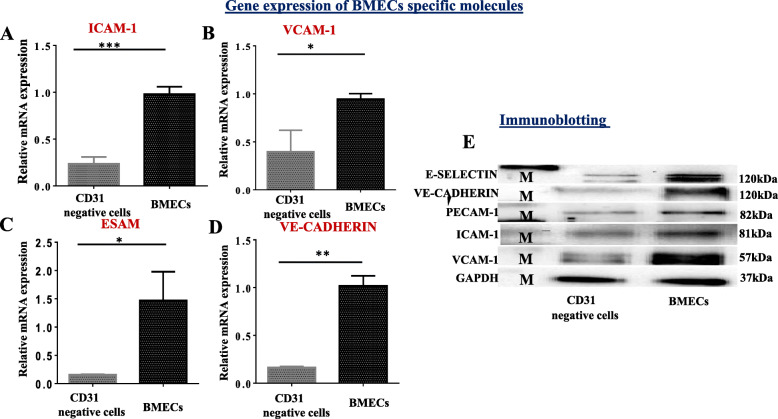
Characterization of primary bone marrow endothelial cells by RT-QPCR and immunoblotting: **a–d** the relative mRNA expression of primary bone marrow endothelial cells compared to CD31 microbead-negative selected cells. The bar chart indicated the fold of the gene expression of primary BMECs compared to the CD31 microbead-negative selected cells. The fold change is quantified by the Pitfall method, followed by a Student’s test comparison between the CD31 microbead-negative selected cells and endothelial cells. *P* value ≤0.05 considered being statistically significant. **e** The immunoblotting analysis of primary bone marrow endothelial after 7 days of cultured further substantiates the purity of endothelial cells with protein ladder (*M*) (*N*= 3 indicates independent experiments for RT-qPCR and *N*=2 for immunoblotting with six mice/group (12 femora and 12 tibias). *p<0.05 **p<0.01, and ***p<0.001

We evaluated gene molecules’ expression specific for endothelial cells using real-time qPCR and immunoblotting, respectively. The relative messenger RNA result shows that the cells isolated highly expressed for ICAM-1, VCAM-1, ESAM, and VE-cadherin, respectively, compared to the CD31 microbead-negative selected cells as shown in Fig. 4a–d. Furthermore, the immunoblotting shows an increase in the expression of E-selectin, VE-cadherin, PECAM-1, ICAM-1, and VCAM-1, respectively, as shown in Fig. 4e. Since these gene molecules are precisely expressed by endothelial cells from other tissues, it indicates the cells isolated are characteristics of bone marrow endothelial cells.

### Certification of primary bone marrow endothelial cells by immunofluorescence staining

**Fig. 5:**
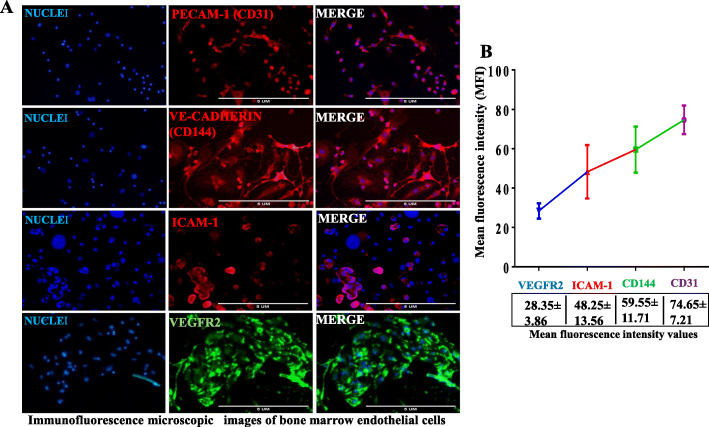
Identification of primary bone marrow endothelial cells by immunofluorescence staining: **a** anti-CD31 (PECAM-1), anti-CD144 (VE-cadherin), anti-CD106 (ICAM-1), and anti-VEGFR2—the cell shows the expression of these endothelial cell-specific molecules. **b** The mean fluorescence intensity for endothelial cells particular marker in the graph was relatively quantified by ImageJ, which shows the order of the endothelial cell-specific markers (PECAM-1> VE-cadherin > ICAM-1 > VEGFR2, *N* = 3 experimental repeats with three mice/group (6 femora and 6 tibias), scale bar 5μm, ×20 magnification

To certified the purity of the primary bone marrow endothelial cells, the cells were cultured in a 48-well plate, after 60–70% of the confluence is achieved then tested against endothelial cell-specific markers, which includes anti-CD31 (PECAM-1), anti-VE-cadherin (CD144), anti-CD106 (ICAM-1), and anti-VEGFR-2, respectively. The cell shows these endothelial cell markers; PECAM-1, VE-cadherin, ICAM-1, and VEGFR2, respectively, as shown in Fig. 5a. The mean fluorescence was quantified using ImageJ software on three experimental repeats, which reveals the expression of the endothelial cell-specific markers in order PECAM-1> VE-cadherin > ICAM-1> VEGFR2, as shown in Fig. 5b. Therefore, these findings confirmed that the cells isolated are precisely bone marrow endothelial cells.

#### The bone marrow endothelial cells’ response to tumor necrosis factor-alpha

**Fig. 6:**
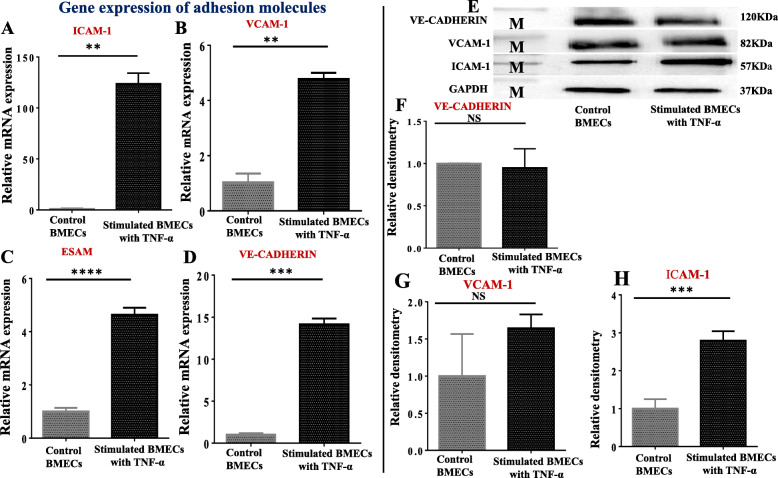
Induction of primary bone marrow-derived endothelial cells by tumor necrosis factor-alpha and quantify by RT-qPCR and immunoblotting. **a**, **b** The relative mRNA expression molecule of intracellular adhesion molecule -1 (ICAM-1) and vascular adhesion molecule -1 (VCAM-1). **c**, **d** The mRNA expression of endothelial junction adherent molecules (ESAM and VE-cadherin) using beta-actin and GAPDH as reference genes. **e** Western blot imaging of proteins with protein ladder (M**). f–h** The relative densitometry of VE-cadherin, VCAM-1, and ICAM-1 using GAPDH as endogenous gene ImageJ software. The bar chart indicates the mean± SD by the Student’s *t* test, (*N* = 3 experimental repeats for 3 mice per group (RT-qPCR and *N*=2 immunoblotting six mice per group). *p< 0, **p<0.01, ***p<0.001, ****p<0.0001, and NS not significant

**Fig. 7:**
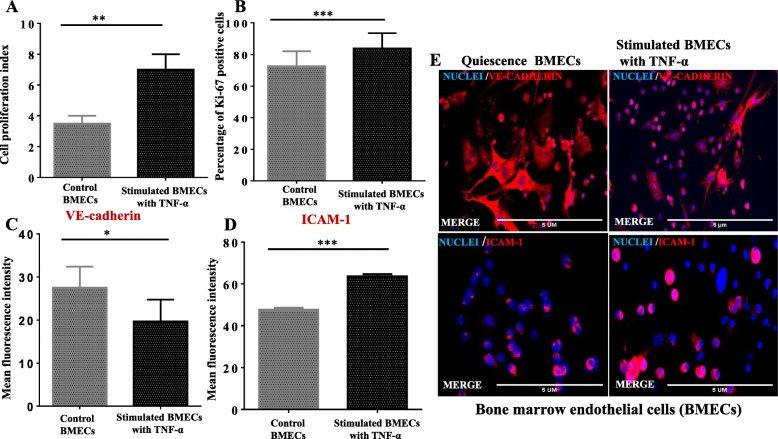
**a**, **b** Bone marrow endothelial cell proliferation Ki-67 by flow cytometry analysis and hemocytometer. **c**, **d** The mean fluorescence of VE-cadherin and ICAM-1 in both quiescence and stimulated BMECs quantified by ImageJ presented in a bar chart with mean± SD. **e** The merged immunofluorescence staining of both quiescence BMECs and treated endothelial cells with TNF-α. Scale bar = 5μm, ×20 magnification, *N*= 3 experimental repeats 3 mice per group, *p< 0.05, **p<0.01, and ***p<0.001

Briefly, the bone marrow endothelial cells culture for 7 days and treated with recombinant tumor necrosis factor-alpha (10ng/ml) for 48 h and untreated cells with 2% FBS in PBS solution. Delta-delta threshold values determined the relative mRNA expression. The bone marrow endothelial cells’ response to tumor necrosis factor-alpha with the increase in fold change of ICAM-1 (122.7 ± 9.93) and VCAM-1 (3.744±0.339), VE-cadherin (13.4 ±0.8179), and ESAM (3.631±0.127) respectively, as shown in Fig. 6a–d (*p*< 0.05) versus the unstimulated BMECs. The immunoblotting results also confirmed an increase in protein expression of ICAM-1 (Fig. 6h, *p*<0.05), but relatively no statistical significance for VCAM-1 and VE-cadherin respectively as compared to the unstimulated BMECs (*p*> 0.05) as shown in Fig. 6f, g). The immunofluorescence staining also confirmed an increase in protein expression of ICAM-1 and decrease protein expression of VE-cadherin compared to the control, as shown in Fig. 7c–e, *p*<0.05). Also, the bone marrow endothelial cells respond to TNF-α by increases in number, as shown in Fig. 7a, b, *p*< 0.05). This data indicates that TN-α enhances the proliferation and growth of bone marrow endothelial cells, as well as the upregulation of adhesion molecules in vitro studies.

## Discussion

This study outlines the step-by-step process of extracting bone marrow endothelial cells from mice long bones. Since bone marrow endothelial cells are a critical component of the sinusoidal-vascular niche in the bone marrow microenvironment, there are no established cell lines for in vitro studies of bone marrow endothelial cells, which respond to inflammatory stimuli and promote hematopoietic stem cell regeneration. The morphological identification of the endothelial cells is a critical step in the process of demonstrating a functional vascular network that is associated with proliferation and differentiation, followed by elongation, and assembled to a capillary-like lumen/linear cord-like vessels forming an infusible vascular tube, as described in published literature [22–24]. The amboidal like the shape of the primary bone marrow endothelial cells is seen on the third-day post-culture, the atypical spindle-like structure appeared on day 7 and is like a cobblestone of the cells after 100% confluence. The matrigel assay shows the perfusable capillary-like lumen and tubing formation, which is a characteristic of an endothelial cell structure which is consistent with published articles [13, 25, 26]. The bone marrow microenvironment consists of three vascular networks: arterioles, transitional, and sinusoids. The endothelial cells are enclosed to sinusoid-vascular that form the endothelial-vascular niche in the hematopoietic stem cell that supports the maintenance and retains the function of the stem cell niches. Besides, within the perivascular slots of the HSC, they are interconnected by endothelial adherent molecules, including VE-cadherin and ESAM as well as endothelial immunoglobulin-like adhesion molecules including PECAM-1 (CD31), E-selectin, ICAM-1 (CD106), and VCAM-1 (CD105) [5, 9, 27, 28]. Therefore, validation of primary bone marrow endothelial cells using these molecules will be essential in the isolation of pure endothelial cells from mice bone marrow. The cells isolated demonstrate the characteristic expression of these adhesion molecules. We further verified by expression of the relative messenger RNA and the protein expression of these adhesion molecules, respectively. Also, primary bone marrow endothelial cells were certified by immunofluorescence staining and show features of these adhesion molecules in sequential order PECAM-1, VE-cadherin, ICAM-1, and VEGFR2, respectively. Finally, we evaluated the response of bone marrow endothelial cells with recombinant tumor necrosis factor-alpha. Our results show that bone marrow endothelial cells increase in proliferation and expression of adhesion molecules, respectively. Also, vascular endothelial cadherin’s protein expression decreases with the change in the cells’ typical shape [29–32].

### Comparison of current protocols with established literature

We developed the current method due to the scarcity of a fluorescence-activated cell-sorting machine to used already established protocols [13–16]. We modified a new strategy of isolation of bone marrow endothelial cells from long mouse bones. Our protocols adopt the cultured marrow-derived adherent cells using CD31 microbeads followed by a single magnetic suspension [17]. In doing, our goals were to develop a simple strategic approach that (1) enabled isolation of the cultured marrow-derived adherent cells without enzymatic digestion, (2) isolation of bone marrow endothelial cells without the needs of discontinuous gradient centrifugation on Percoll and fluorescence-activated cell-sorting (FACS), (3) production of viable bone marrow endothelial cells and minimizing contamination of fibroblast and mononuclear phagocytes, and (4) unlocking the challenges for researchers interest in studying endothelial cells with the bone marrow microenvironment for stem cell research. Using the literature from some of the previously established methods, we developed a new approach to accomplish these goals. We used the combination of endothelial cell-specific markers used in cell-sorting as an advantage to isolate pure bone marrow endothelial cells. Using this protocol, researchers can isolate highly pure endothelial cells from the bone marrow with minimal contaminations of other marrow-adherent cells. The isolated cells can be used to evaluate the bone marrow microenvironment and related studies using the mouse model. However, the current method cannot be used for in vivo studies, and fluorescence-activated cell-sorting still has to be utilized.

### The importance and future application of bone marrow endothelial cells

Researchers can now use this novelty method to determine the molecular or gene fingerprint of bone marrow endothelial cells and identify the vascular mechanisms governing the bone marrow microenvironment. Researchers can also use this method to understand the role of the endothelial-vascular niche in inflammation, angiogenesis, and its influence in supporting the interaction between the hematopoietic stem cell (HSC) steady-state malignant process in the mouse model. Lastly, the researcher can explore this method to initiate endothelial cell stem transplantation in a mouse model and improve endothelial stem cell research’s practicality.

## Conclusion

We established a novel method for the isolation of primary bone marrow endothelial cells from mice bone marrow, which can be used to evaluate endothelial-vascular niche in vitro studies. Furthermore, bone marrow endothelial cell transplantation will become clinically applicable soon using the mouse model. We suggest the following factors must be considered when isolating these cells.

### Pre-requisites processing

1. Before isolation, boil all instruments, air dry in the oven for at least 30 min, and add gauze to autoclave.
2. Prepare fresh DPBS solution with PH 7.2–7.6 and 0.5 M EDTA and sterilize.
3. Sterilize 0.5-ml Nest microtubes and 1.5-ml Eppendorf tubes for density centrifugation of the bone marrow cell from the mice bones.
4. Prepare sterile 1-mm EDTA/DPBS and harvesting buffer solution kept at 4°C for 30 days only.
5. Pre-coat the dish with rat-tail collagen overnight at 37°C in the incubator, wash three times with sterile DPBS, and air dry for 30 s before pouring the endothelial cell growth media.

### Isolation

1. Try to spend less time harvesting the bones from the mice, wash bones with sterile DPBS, and a final wash with DPBS containing 1-mm EDTA to prevent blood clotting and clumping of the cells.
2. Use a 5-ml syringe needle to perforate the center of the 0.5-ml tube and invert the cut edge of the bones [maximum (4) 2 tibias, 2 femora], and suspend the pellet with DPBS/1 mm EDTA.
3. Incubate the bone marrow cells, not more than 24 h.
4. Change endothelial cell medium supplement with growth factors after 3–4 days to maintain the cell numbers.

### Limitation

1. Getting enough cells involves many mice (more than 12 mice/group), especially for immunoblotting techniques.
2. The cell passage number should not exceed two passages (passages 0 and 1 recommended). In more than three passages, the cells are prone to fungal infection and cell drift.
3. Contamination of primary bone marrow endothelial cells is common with macrophages, fibroblasts if incubated cells for more than 24 h. To avoid that, try to harvest cells less than a 24-h incubation and repeat passage for the cells twice via the column.
4. The endothelial growth factor media are susceptible to infection and have an excellent working sterile environment key to get optimal results.
5. The primary bone marrow endothelial cells have difficulties attached to the glass slide for immunofluorescence staining for this procedure using 48 or 96 wells to get optimal results.

## Supplementary Information

**Additional file 1: Figure S1.** supplementary figure to figure 5: (A) Identification of primary bone marrow endothelial cells; Alexfluro 594 fluorescence RED and Alexfluro 488 fluorescence GREEN were used as secondary antibodies, respectively. These secondary antibodies were stained independently without the primary antibodies respective. The results illustrate the secondary antibodies do not cross-react with primary antibodies and don’t bind unnecessarily. Ns= 3 experimental repeats with three mice per group. Scale bar = 5μm , 20x magnifications. (PPTX 73 kb)

## Data Availability

All relevant data in the study are included in the article; further inquiries can be directed to the corresponding authors.

## Abbreviations

BMECs: Bone marrow endothelial cells
ECs: Endothelial cells
1 (ICAM-1): Intracellular adhesion molecule
1 (VCAM-1): Vascular adhesion molecule
ESAM: Vascular endothelial selectin adhesion molecules
VE-cadherin: Vascular endothelial cadherin molecule
EDTA: Ethylenediaminetetraacetic acid
CD: The cluster of differentiation
DPBS: Dulbecco’s phosphate buffer solution
GAPDH: Glyceraldehyde-3-phosphate dehydrogenase
VEGFR2: Vascular endothelial cells growth factor receptor
ESAM: Endothelial selective adhesion molecule
VE-Cadherin: Vascular endothelial cadherin
