# Supplementary figures and images for: A novel strategy for isolation of mice bone marrow endothelial cells (BMECs)
Source: Stem Cell Res Ther. 2021 May 3;12:267. doi: 10.1186/s13287-021-02352-3 (PMC8091666; doi:10.1186/s13287-021-02352-3)

## Slide 1
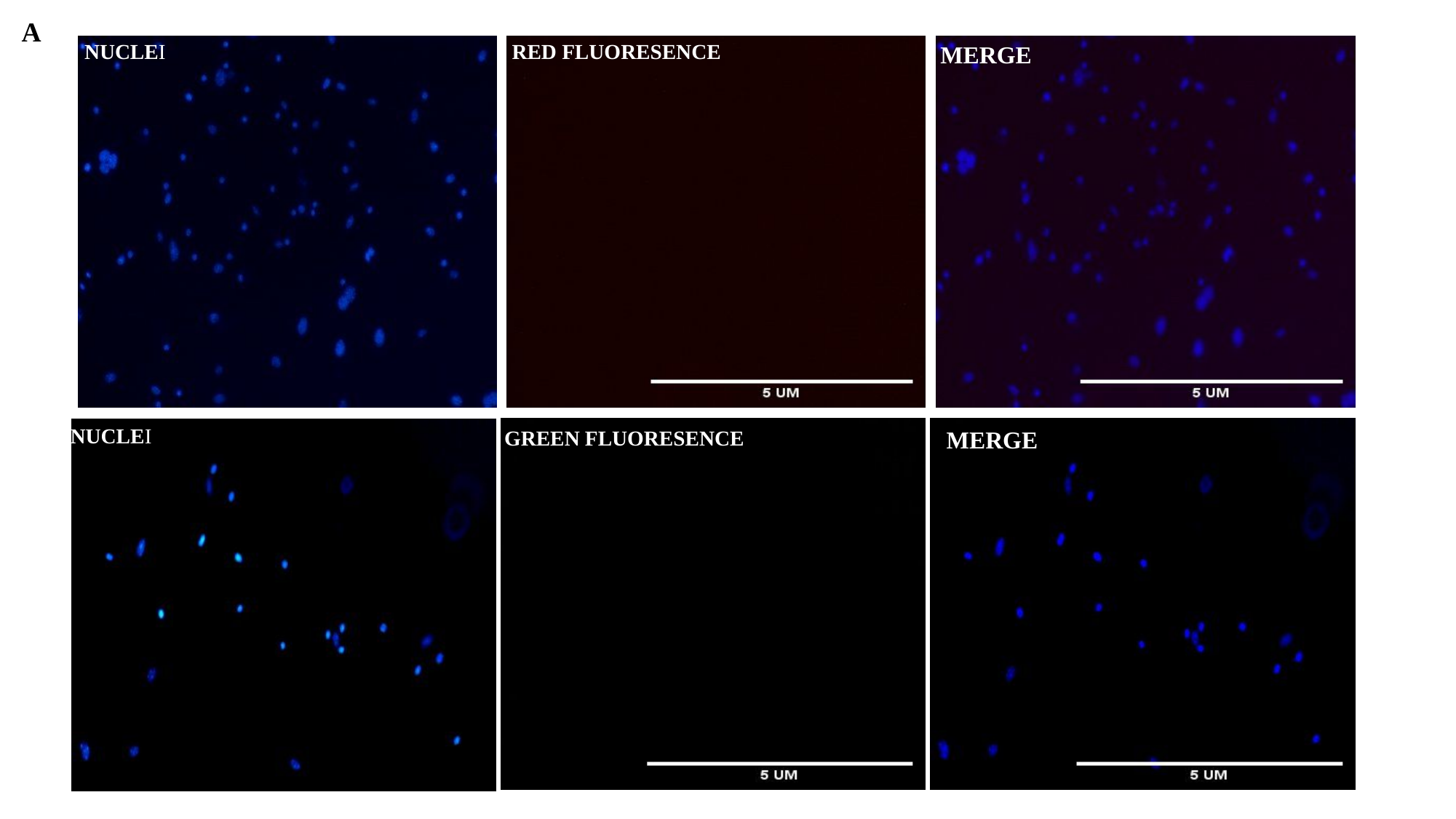

A
NUCLEI
RED FLUORESENCE
NUCLEI
GREEN FLUORESENCE
MERGE
MERGE

Supplement: Supplementary file 1 — Additional file 1: Figure S1. supplementary figure to figure 5: (A) Identification of primary bone marrow endothelial cells; Alexfluro 594 fluorescence RED and Alexfluro 488 fluorescence GREEN were used as secondary antibodies, respectively. These secondary antibodies were stained independently without the primary antibodies respective. The results illustrate the secondary antibodies do not cross-react with primary antibodies and don’t bind unnecessarily. Ns= 3 experimental repeats with three mice per group. Scale bar = 5μm , 20x magnifications. (PPTX 73 kb) [file 13287_2021_2352_MOESM1_ESM.pptx]
